# Association between *Ureaplasma urealyticum* colonization and bronchopulmonary dysplasia in preterm infants: a systematic review and meta-analysis

**DOI:** 10.3389/fped.2024.1436568

**Published:** 2024-08-08

**Authors:** Xianhong Chen, Xunbin Huang, Qiujing Zhou, Houxin Kang, Huixian Qiu, Lindong Shi, Hong Tang, Shujuan Zeng

**Affiliations:** ^1^ Shenzhen Clinical Medical College, Guangzhou University of Chinese Medicine, Shenzhen, Guangdong, China; ^2^ Division of Neonatology, Longgang District Central Hospital of Shenzhen, Shenzhen, Guangdong, China; ^3^ Neonatal·Child Critical Child Health Care Division, The Central Hospital of Enshi Tujia and Miao Autonomous Prefecture, Enshi Tujia and Miao Autonomous Prefecture, Hubei, China; ^4^ Division of Neonatology, Shenzhen Yantian District People’s Hospital, Shenzhen, Guangdong, China

**Keywords:** *Ureaplasma urealyticum*, bronchopulmonary dysplasia, preterm infants, association, meta-analysis

## Abstract

**Background:**

Bronchopulmonary dysplasia (BPD) is the most prevalent chronic lung disease in preterm infants. Studies have shown that *Ureaplasma urealyticum* (UU) infection is linked to its pathogenesis. However, it remains controversial whether UU colonization in preterm infants increases the risk of developing BPD.

**Objective:**

This study aimed to conduct a systematic review and meta-analysis to summarize the correlation between UU and BPD.

**Methods:**

We searched PubMed, Embase, the Cochrane Library, Web of Science, Wanfang Database, Chinese National Knowledge Infrastructure Database, Chinese Science and Technique Journal Database, and the China Biology Medicine disc from their inception to March 15, 2024. We included cohort and case-control studies investigating the association between UU infections and BPD in preterm infants, adhering to the Preferred Reporting Items for Systematic Reviews and Meta-Analyses (PRISMA) guidelines. The Newcastle-Ottawa Scale was used for quality assessment. The outcome was defined as the continued need for oxygen or respiratory support at 28 days after birth (BPD28) or at 36 weeks postmenstrual age (BPD36). Considering the potential publication bias in observational studies, we used a random-effects meta-analysis model, assessed heterogeneity (I^2^), performed subgroup analyses, evaluated publication bias, and graded the quality of evidence.

**Results:**

The meta-analysis included 36 cohort studies encompassing 5,991 participants. Among these, 20 reported on BPD28, 13 on BPD36, and 3 on both. The results indicated a significant association between UU colonization and BPD28 (odds ratio (OR): 2.26, 95% confidence interval (CI): 1.78–2.85, *P* < 0.00001, 23 studies, very low certainty of evidence) and BPD36 (OR: 2.13, 95% CI: 1.47–3.07, *P* < 0.0001, 16 studies, very low certainty of evidence).

**Conclusion:**

There is a correlation between UU colonization and the development of BPD in preterm infants. Future research should prioritize well-designed, large-scale, high-quality randomized controlled trials (RCTs) to comprehensively assess the risk of BPD in neonates following UU infection and to provide stronger evidence for clinical screening and prevention strategies to improve the prognosis of affected newborns.

**Systematic Review Registration:**

https://www.crd.york.ac.uk/, identifier (CRD42024524846).

## 1 Introduction

Bronchopulmonary dysplasia (BPD) is the most prevalent and severe chronic lung disease in preterm births (1). BPD affects at least one in four infants weighing less than 1,500 grams (g), with incidence increasing as birth gestational age and weight decrease (1). The primary clinical features of BPD include chronic dependence on oxygen and dysregulated lung growth. BPD is associated with increased mortality, respiratory morbidity, neurodevelopmental deficits, pulmonary hypertension, and cardiac dysfunction (2, 3). BPD not only severely impacts the quality of life of affected infants but also escalates economic, psychological, and social burdens, marked by prolonged intensive care stays, requisite home oxygen therapy post-discharge, and frequent rehospitalizations due to pulmonary deterioration (4–6). A comprehensive study in Spain demonstrated that BPD substantially increases healthcare costs within the first two years of a preterm infant's life (7). A Canadian survey indicated that BPD and its complications impose significant financial strains and broadly detract from the quality of life of preterm infants born at or before 28 weeks of gestation (8).

BPD is influenced by numerous factors, yet its precise etiology remains elusive. Increasing evidence suggests that intrauterine infections increase the risk of BPD. The predominant pathogen in intrauterine infections is *Ureaplasma urealyticum* (UU), with the risk of infection escalating as gestational age decreases (9–11). UU, an opportunistic pathogenic microorganism, adheres to human epithelial and germ cells and can colonize the mucosal surfaces of the respiratory tract in both adults and infants (12). One study reported that vertical transmission of UU occurs in 45%–66% of neonates, with higher rates observed in preterm infants, and that many unexplained preterm births are due to UU infection (13). Furthermore, UU has been shown to cause neonatal lung injury by intensifying the inflammatory response, resulting in prolonged and dysregulated inflammation and subsequent lung injury in preterm infants (14).

In 1988, Cassell et al. (15) were the first to report the association between UU infection and both lung injury and death. Despite 36 years passing since the initial report of neonatal respiratory Ureaplasma colonization associated with BPD, controversy persists regarding its role in causing BPD. Currently, there are four meta-analyses on the association between UU and BPD (14, 16–18). Previous systematic reviews and meta-analyses have demonstrated a significant association between UU colonization and BPD diagnosed 28 days after birth (14, 16, 17). However, debate continues regarding whether this association extends to BPD diagnosed at 36 weeks postmenstrual age (14, 16–18). Furthermore, inconsistencies in applying uniform criteria for inclusion across previous studies may have influenced the results. Consequently, whether UU colonization can increase the incidence of BPD remains contentious.

Since two systematic reviews and meta-analyses were published in 2014 (17, 18), there have been no subsequent updates in meta-analyses regarding the correlation between UU colonization and BPD over the past decade. The interpretation of the available data may be subject to change due to inconsistency in diagnostic criteria regarding BPD in previous studies and the fact that recently published studies have not been included in any previous meta-analyses to date. In this context, assessing the available evidence and qualitatively and critically evaluating the existing gaps is essential. Consequently, we conducted a systematic evaluation and update of meta-analyses examining the correlation between UU colonization and BPD. The primary objective of this review was to ascertain whether UU colonization correlates with the development of BPD in preterm infants, considering standardized diagnostic criteria. The secondary objective was to evaluate the influence of factors such as gestational age, birth weight, sample types, and testing methods on the correlation between UU and BPD.

## 2 Methods

### 2.1 Registration

This study conforms to the Preferred Reporting Items for Systematic Reviews and Meta-Analysis (PRISMA) consensus guidelines (19) (Supplementary File S1). The study has been registered with the International Prospective Register of Systematic Reviews (PROSPERO; registration number: CRD42024524846). The systematic evaluation, being a secondary analysis of existing literature, was deemed not to require ethical review.

### 2.2 Data sources and search strategy

The search strategy was implemented across eight electronic databases, including PubMed, Embase, Cochrane Library, Web of Science, Wanfang Database, Chinese National Knowledge Infrastructure Database (CNKI), Chinese Science and Technique Journal Database (VIP), and China Biology Medicine disc (CBM). The search spanned from the inception of the databases until March 15, 2024, imposing no restrictions on language or publication date. Search terms including “Bronchopulmonary Dysplasia”, “Ureaplasma urealyticum” or “Ureaplasma” were used, combining Medical Subject Headings (MeSH) and free-text terms. A detailed overview of the search strategy is available in Supplementary File S2. We also manually checked references in the included studies.

### 2.3 Eligibility criteria

The inclusion criteria for study selection included: (1) Population: Preterm infants under 37 weeks of gestational age admitted to the neonatal ward, with stays long enough to observe BPD outcomes; (2) Exposure and risk factor: Preterm infants with UU colonization, defined as detection of UU via polymerase chain reaction (PCR) analysis or culture from infant specimens; (3) Comparison: Preterm infants uninfected with UU at or before BPD diagnosis; (4) Outcome: BPD diagnosis was based on either the 2001 or 2018 National Institute of Child Health and Human Development (NICHD) diagnostic criteria or the criteria proposed by Jensen et al. in 2019 (20–22). There are two definitions of BPD: the first definition considers preterm infants who still require oxygen or respiratory support at 28 days postnatal (20), and the second defines preterm infants who still need oxygen or respiratory support at 36 weeks postmenstrual age (21, 22). The presence of abnormal x-ray findings is not necessary; (5) Study design: cohort study or case-control study. Supplementary File S3 contains the details of the eligibility criteria and outcome definitions.

Exclusion criteria: (1) Studies with imprecise designs; (2) Studies containing duplicate or overlapping data; (3) Non-original research, such as conference abstracts, clinical trial registries, reviews, systematic reviews, meta-analyses, guidelines, animal experiments, and case reports; (4) Incomplete data.

### 2.4 Study selection

All relevant articles were import into EndNote X9 reference management software to remove any duplicates. Two researchers (X.C. and X.H.) independently screened the literature using predefined criteria. Initially, we evaluated the titles and abstracts of the articles to exclude those not meeting the criteria, followed by a detailed review of the full texts to further exclude irrelevant studies. Disagreements between the researchers were resolved through discussion, with a third researcher (S.Z.) making a decision when necessary.

### 2.5 Data extraction

Data extraction for the study was conducted using a standardized Microsoft Excel data extraction form. Two review authors (X.C. and X.H.) independently extracted the data and cross-checked their findings. Disagreements will be resolved through discussion, or if necessary, by consulting a third author (H.T.) to reach a consensus. If necessary, authors of individual studies will be contacted to obtain additional data or clarify results. The following data were collected: (1) Basic information (author name, publication year, country of study, study period and study design); (2) Characteristics of the included population (birth gestational age and birth weight of preterm infants, total number of participants, number lost to follow-up); (3) Specimen type, time of collection, and laboratory method of microbe identification (culture and/or PCR); (4) Results (diagnostic criteria for BPD, number of cases in exposed and non-exposed groups, and total number of infants in each of the two groups).

### 2.6 Risk of bias and grade certainty assessment

The study encompassed observational cohort or case-control studies. The risk of bias was evaluated by two researchers (X.C. and X.H.) utilizing the Newcastle-Ottawa Scale (NOS), comprising three domains: selection, comparability, and outcome (23). Any disagreements were resolved through group discussions. Supplementary File S4 outlines guidelines for assessing grading method quality. The quality of evidence from included studies was assessed using the Grading of Recommendations Assessment, Development and Evaluation method (GRADEpro Guideline Development Tool, gradepro.org) (24).

### 2.7 Statistical analysis

We performed a meta-analysis of the literature that reported BPD data in both UU-infected and control groups. Raw data were analyzed with statistical software Review Manager 5.4 (the Cochrane Library) and Stata 17 (Stata Corp). Forest plots for visualizing results of meta-analysis. The impact of UU on BPD was quantified using odds ratio (OR). A 95% confidence interval (CI) was calculated. The Cochran's Q and inconsistency index (*I^2^*) test were employed to assess the heterogeneity among the study results. Significant heterogeneity was indicated if *I^2^* was ≥50% or if the *p*-value of Cochran's Q was <0.05. Given the predominantly prospective or retrospective nature of the included observational studies, significant clinical or methodological differences might exist. Consequently, we conducted the meta-analysis using a random-effects model. Subgroup analyses were conducted based on the study continent, birth gestational age, birth weight of preterm infants, sample collection site, and testing method to explore sources of heterogeneity and assess the impact of these factors on the results (Supplementary File S5). We systematically excluded each study individually to determine its influence on the pooled effect estimates. When the number of included studies exceeded 10, potential publication bias was assessed through visual inspection of funnel plots and quantified using Egger's test.

## 3 Results

### 3.1 Literature search and study selection

**Figure 1:**
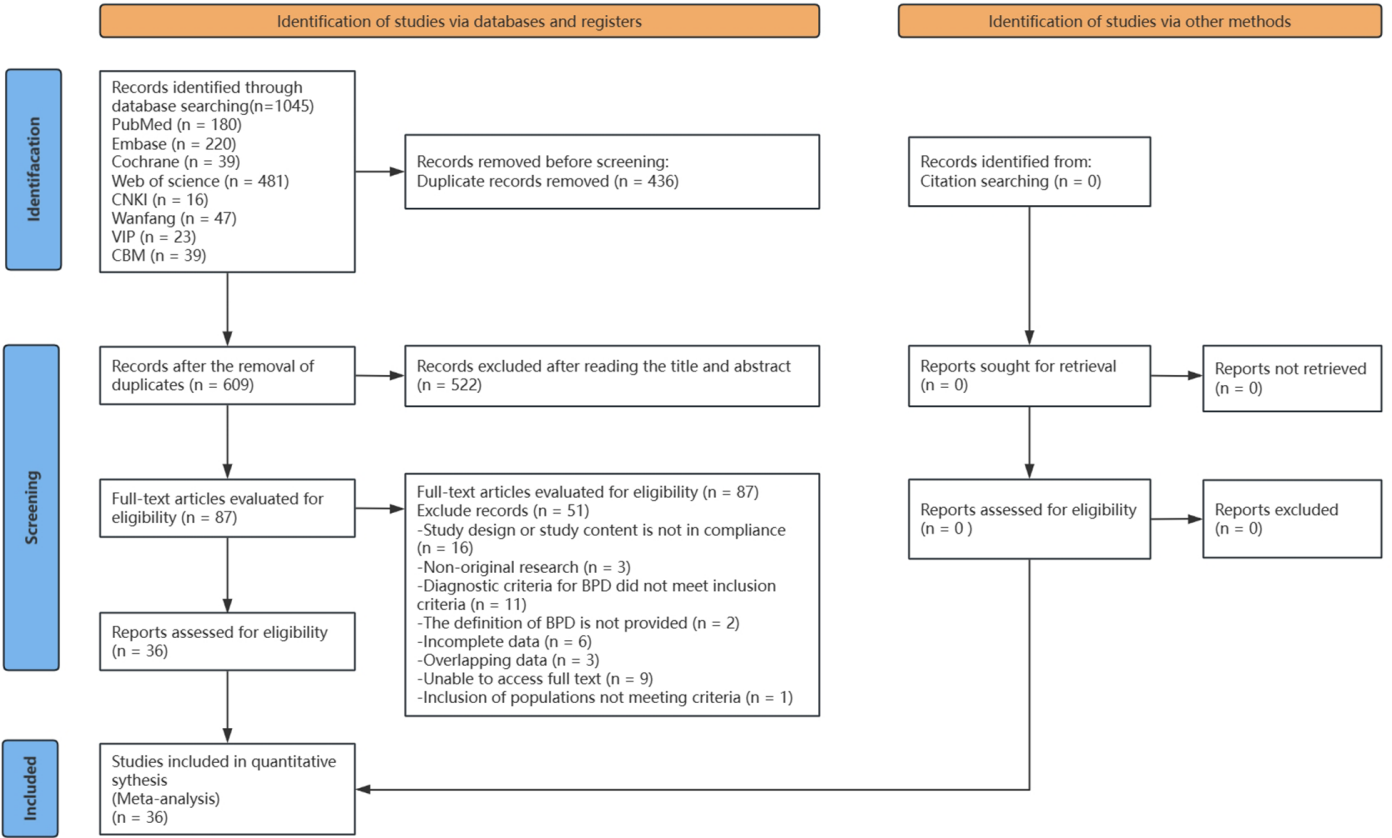
PRISMA flow diagram shows the systematic search of the literature.

Initially, we identified 1,045 relevant articles, excluding 436 due to duplicates. We then screened 609 articles based on exclusion and inclusion criteria by reviewing titles and abstracts, excluding 522. The remaining 87 articles underwent full-text review. After full-text screening, 51 articles were eliminated for not meeting the inclusion criteria (Supplementary File S6). Ultimately, 36 publications (15, 25–59) were included in this meta-analysis (Figure 1).

### 3.2 Characteristics of the included studies

The 36 included studies (15, 25–59) analyzed a total of 5,991 participants, involving between 32 and 585 samples, across 13 countries. All included studies were cohort studies (52.78% prospective, 47.22% retrospective). Characteristics of all studies are displayed in Table 1. The study population predominantly consisted of preterm infants or those with low birth weight.

**Table 1:** Characteristic of the included studies.

Study no.	Study ID	Study period	Study design	Country	Study population	GA (weeks), mean (SD), median (IQR) or range	BW (grams), mean (SD) or rang	Outcomes	Specimen types	Detection methods	Sample size
1	Zheng Huaiwu (26)	2017.01–2021.12	Prospective cohort	China	Preterm infants with <34 weeks	32.45 (1.64)	1,812.79 (461.81)	①	Pharyngeal secretions or Endotracheal aspirates	PCR	182
2	Fan Xufang (27)	2019.03–2022.02	Retrospective cohort	China	28–32w AND <1,500 g	29.99 (1.00)	1,307.06 (110.87)	①	Endotracheal aspirates	PCR	327
3	Zhong Linping (25)	2019.06–2022.03	Retrospective cohort	China	Gestational age <28 weeks or birth weight <1,000 g	27.29 (0.15)	937.32 (29.27)	②	Endotracheal aspirates	PCR	82
4	Wei Hongling (28)	2018.01–2019.12	Retrospective cohort	China	<32w	28.65 (0.00)	1,226.71 (313.78)	①	Respiratory secretions	PCR	105
5	Chen Jing (30)	2019.01–2020.08	Retrospective cohort	China	<32w	28.74 (1.81)	1,197.47 (290.14)	①	Endotracheal aspirates or nasopharyngeal aspirates	PCR	217
6	Chen Xianrui (29)	2017.06–2018.05	Retrospective cohort	China	<37w	31.79 (3.21)	1,829.09 (675.74)	①	Endotracheal aspirates	PCR	194
7	Sun, Tong (31)	2019.01–2021.01	Retrospective cohort	China	Preterm infants born in our hospital and hospitalized in our department and gestational age no more than 32 weeks, birth weight no more than 2,000 g	30.02 (0.00)	1,286.27 (276.70)	②	Lower respiratory tract secretion, gastric fluid and urine	PCR	291
8	WU Yongfang (33)	2017.04–2019.11	Retrospective cohort	China	Preterm infants born at ≤32 weeks of gestational age	29.10 (0.00)	1,233.26 (288.19)	①	Pharyngeal secretions	PCR	399
9	SUN, Qin (32)	2018.03–2018.09	Retrospective cohort	China	Preterm infants born at <32 weeks of gestational age	29.69 (0.00)	1,287.29 (319.77)	①	Respiratory secretions	PCR	147
10	Chun, Jiyoung (35)	2012.01–2016.12	Retrospective cohort	South Korea	Preterm infants delivered at <32 weeks’ gestational age (GA)	27.16 (2.35)	Undescribed	①	Tracheal aspiration (intubated infants) or nasopharyngeal swabs (nonintubated infants)	PCR	245
11	Mahallei, M (34)	2017.03–2018.03	Prospective cohort	Iran	Newborns weighing less than 1,500 g with the gestational age of less than 32 weeks who required intubation within 72 h after birth	28.55 (1.95)	1,099.70 (216.45)	①	Endotracheal secretions	PCR	61
12	ZHANG Dan (37)	2014.12–2015.04	Retrospective cohort	China	VLBWI infants (gestational age ≤32 weeks)	≤32	< 1,500	①	Venous Blood	PCR	80
13	ZHENG Lajie (36)	2011.05–2015.04	Retrospective cohort	China	Preterm infants under 34 weeks’ gestation	30.60 (1.53)	1,303.84 (53.79)	①	Respiratory secretions	PCR	232
14	Chen Ronghua (38)	2013.06–2014.06	Retrospective cohort	China	Preterm infants ≤32 weeks of gestational age, admitted to the NICU within 2 days of birth, and surviving until 36 weeks of corrected gestational age	29.22 (1.63)	1,240.96 (21.11)	②	Pharyngeal or airway secretions	PCR	585
15	Shi Wei (39)	2010.02–2013.02	Retrospective cohort	China	Preterm infants with gestational age ≤34 weeks, age <48 h at admission and hospitalized for more than 2 weeks	30.26 (1.24)	1,439.78 (131.19)	①	Pharyngeal or airway secretions	PCR	207
16	Chen You (40)	2011.10–2013.05	Retrospective cohort	China	Preterm infants <37 weeks gestation	31.46 (2.23)	1,603.43 (385.67)	①	Gastric fluid	PCR	270
17	Liu Fang (41)	2011.01–2012.10	Retrospective cohort	China	Preterm infants admitted to the neonatal unit with an admission age of <12 h, gestational age of 29–36 weeks, and birth weight of 1.3–3.0 kg	29–36	1,300–3,000	①	Gastric fluid	PCR	139
18	Inatomi, T (43)	2007.01–2010.03	Prospective cohort	Japan	Infants with gestational age (GA) <29 w or birth weight <1,000 g	27.42 (0.65)	909.48 (30.28)	①	Gastric fluid	PCR	122
19	BAO Yu (42)	2004.01–2011.06	Retrospective cohort	China	All infants whose gestational age was ≤32 weeks and survived at 36 weeks were included in this study	30.17 (1.45)	1,249.39 (23.48)	①	Pharyngeal or airway secretions	PCR	561
20	Beeton, M. L (44)	Not describe	Prospective cohort	UK	Pre-term (≤34 weeks gestation)	≤34	Undescribed	②	Gastric fluid, tracheal aspirates and lung fluid sample	Culture, PCR	123
21	Pandey, A (45)	2003.06–2005.06	Prospective cohort	India	All infants with gestational age <34 w, weighing <1,800 g who were admitted to the Unit within 24 h of birth and had not received prior antibiotic therapy	30.98 (1.99)	1,222.36 (298.88)	①	Nasopharyngeal or endotracheal aspirates	PCR	100
22	Egawa, Tsuyoshi (46)	2004.04–2006.03	Prospective cohort	Japan	<32W	28.00 (23–31)	1,006 (478–1,810)	②	Umbilical cords, placentas, Tracheal aspiration or GF samples	PCR	118
23	Colaizy, Tarah T (47)	1998.01–2002.12.31	Prospective cohort	America	VLBW infants	26.90 (1.86)	957.09 (242.42)	②	Endotracheal aspirates	PCR	121
24	Aaltonen, R (48)	1996.01–2000.03	Prospective cohort	Finland	Infants born before the 30th week of gestation	27.08 (0.74)	967.37 (15.17)	①②	Bronchotracheal lavage sample	PCR	49
25	Adcock, Kim G (49)	Not describe	Prospective cohort	America	Birth weight less than 1,500 g who required mechanical ventilation for RDS were enrolled into this study with parental consent.	26.74 (0.59)	912.82 (137.57)	②	Tracheal aspirates	Culture	44
26	Mhanna, Maroun J (50)	1996–1999	Retrospective cohort	America	Medical records of all mechanically ventilated VLBW infants,	26.20 (1.70)	737.00 (167.10)	②	Tracheal aspirates	Culture	100
27	Castro-Alcaraz, S (52)	1993.03–2000.04	Prospective cohort	America	Infants weighing <1,500 g and/or <32 weeks’ gestational age over a 12-month period	27.89 (1.18)	1,009.62 (117.11)	①②	Endotracheal, nasopharyngeal, and throat specimens	Culture, PCR	120
28	Ruf, B (51)	1998.01–1999.11	Prospective cohort	Germany	VLBW's preterm infants	28.23 (0.42)	1,047.57 (50.82)	①	Pharyngeal swabs	Culture	74
29	Galetto Lacour, A (54)	1993.06–1995.07	Prospective cohort	Switzerland	Preterm infants less than 32 weeks of gestational age	28.65 (2.31)	1,080.40 (75.32)	①	Endotracheal aspirates or nasopharyngeal aspirates	Culture	50
30	Ollikainen, J (53)	1990.06–1991.12;1993.02–1994.07	Prospective cohort	Finland	<34W	29.52 (0.42)	1,344.84 (37.30)	②	Endotracheal aspirates OR blood	Culture	124
31	Hannaford, K (55)	1993.10–1997.06	Prospective cohort	Australia	Infants of less than 28 weeks of gestational age	<28	Undescribed	①②	Endotracheal aspirates	Culture	112
32	Perzigian, R. W (56)	1994.04	Prospective cohort	America	VLBW infants who required mechanical ventilation at <12 h of age	28.79 (2.29)	1,134.01 (261.37)	②	Endotracheal aspirates	Culture	105
33	Pacifico, Lucia (57)	1993.10–1996.01	Prospective cohort	Italy	Birth weights ≤1,500 g; admission to the NICU within 24 h after birth; evidence on admission of respiratory	27.25 (1.89)	842.13 (115.26)	②	Endotracheal aspirates	Culture	32
34	Da Silva, Orlando (58)	1994.05–1995.05	Prospective cohort	Canada	All infants weighing <1,501 g at birth	27.66 (1.97)	1,036.14 (236.89)	②	Endotracheal aspirates, nasopharyngeal aspirates	Culture, PCR	108
35	Iles, R (59)	Not describe	Prospective cohort	UK	Infants less than 31 weeks’ gestation	27.25 (0.98)	868.25 (20.59)	②	Endotracheal secretions	Culture	40
36	Cassell, G. H (15)	1985.07–1987.06	Prospective cohort	America	Infants with birthweight ≤2,500 g and evidence of respiratory disease within 24 h after birth	Undescribed	≤2,500	①	Endotracheal aspirates	Culture	125

PCR, polymerase chain reaction.

① Preterm infants who still require oxygen or respiratory support at 28th day of age; ② Preterm infants who still need oxygen or respiratory support at 36 weeks postmenstrual age.

For the determination of UU colonization, 32 studies utilized respiratory secretion samples (endotracheal aspirate, nasopharyngeal aspirate, pharyngeal swab, bronchial lavage, and lung fluid specimens) (15, 25–36, 38, 39, 42, 44–59). Additionally, 5 studies analyzed gastric fluid samples (31, 40, 41, 43, 44), 2 studies analyzed blood samples (37, 53), 1 study analyzed urine samples (31), and 1 study focused on umbilical cords and placentas (46). Regarding the methods for detecting UU colonization, 23 studies employed PCR for nucleic acid detection (25–43, 45–48), 10 studies used culture (15, 49–51, 53–57, 59), and 3 studies applied both PCR and culture (44, 52, 58).

Twenty studies assessed BPD outcomes only at 28 days post-birth (15, 26–30, 32–37, 39–43, 45, 51, 54), thirteen studies evaluated BPD outcomes solely at 36 weeks of postmenstrual age (25, 31, 38, 44, 46, 47, 49, 50, 53, 56–59), and three studies assessed BPD outcomes at both 28 days post-birth and 36 weeks postmenstrual age (48, 52, 55). The authors of 19 studies concluded that UU infection was positively associated with BPD (26, 27, 31, 32, 35–39, 42–44, 47, 51, 52, 55–57, 59). The authors of 14 studies concluded that UU infection was not associated with BPD (15, 28–30, 33, 34, 40, 41, 45, 46, 48, 50, 53, 58). The authors of 3 studies concluded that UU infection was possibly associated with BPD (25, 49, 54).

### 3.3 Quality assessment

**Figure 2:**
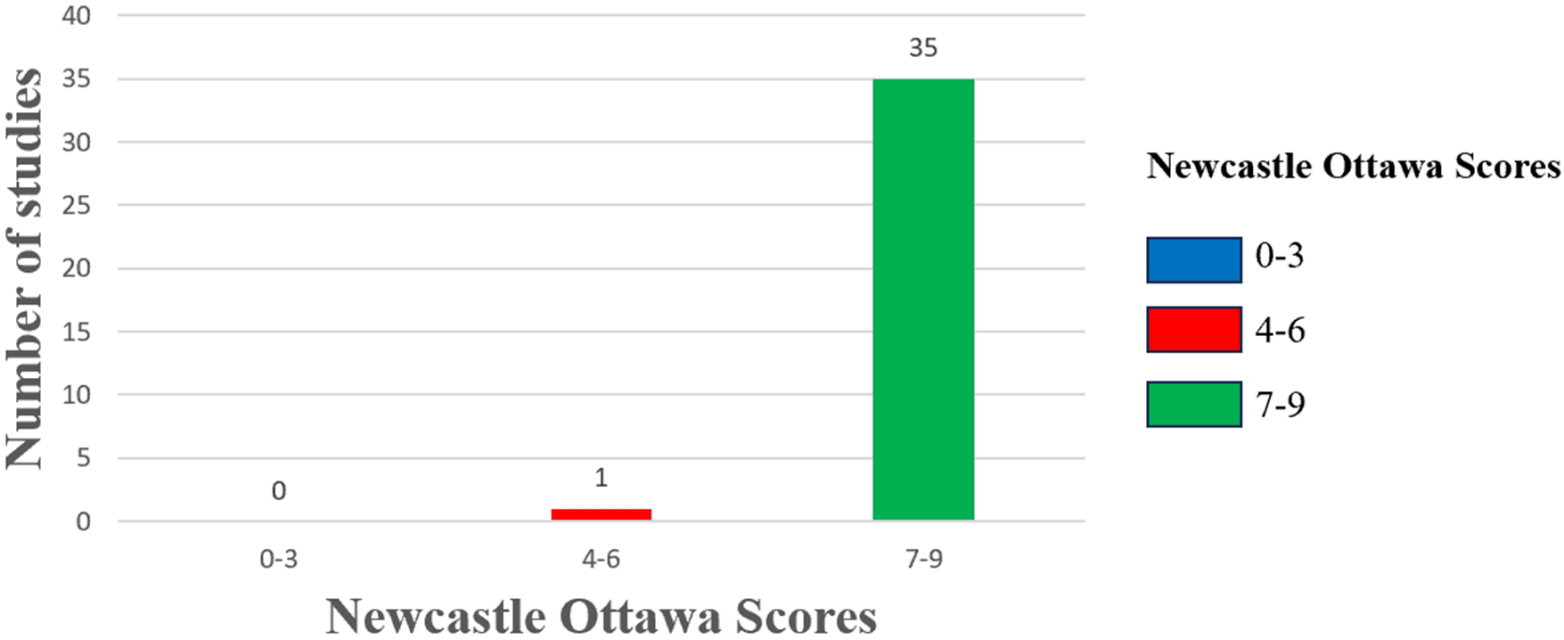
Risk of bias assessment. The column chart shows the Newcastle Ottawa scores along the x-axis divided into 3 groups: high risk of bias (0–3), moderate risk of bias (4–6), and low risk of bias (7–9). The number of studies included in the review with those scores on the y-axis.

We employed the NOS scale to assess the quality of all included studies, categorizing them as high, medium, or low quality following a comprehensive evaluation. The studies analyzed were rated between 6 and 9 on the NOS scale, with one study classified as moderate quality and the remaining thirty-five as high quality (Figure 2). Supplementary File S7 details the specific quality assessment results for each study.

### 3.4 Meta-analysis

#### 3.4.1 Association between *U. ureaplasma* Colonization and BPD28

**Figure 3:**
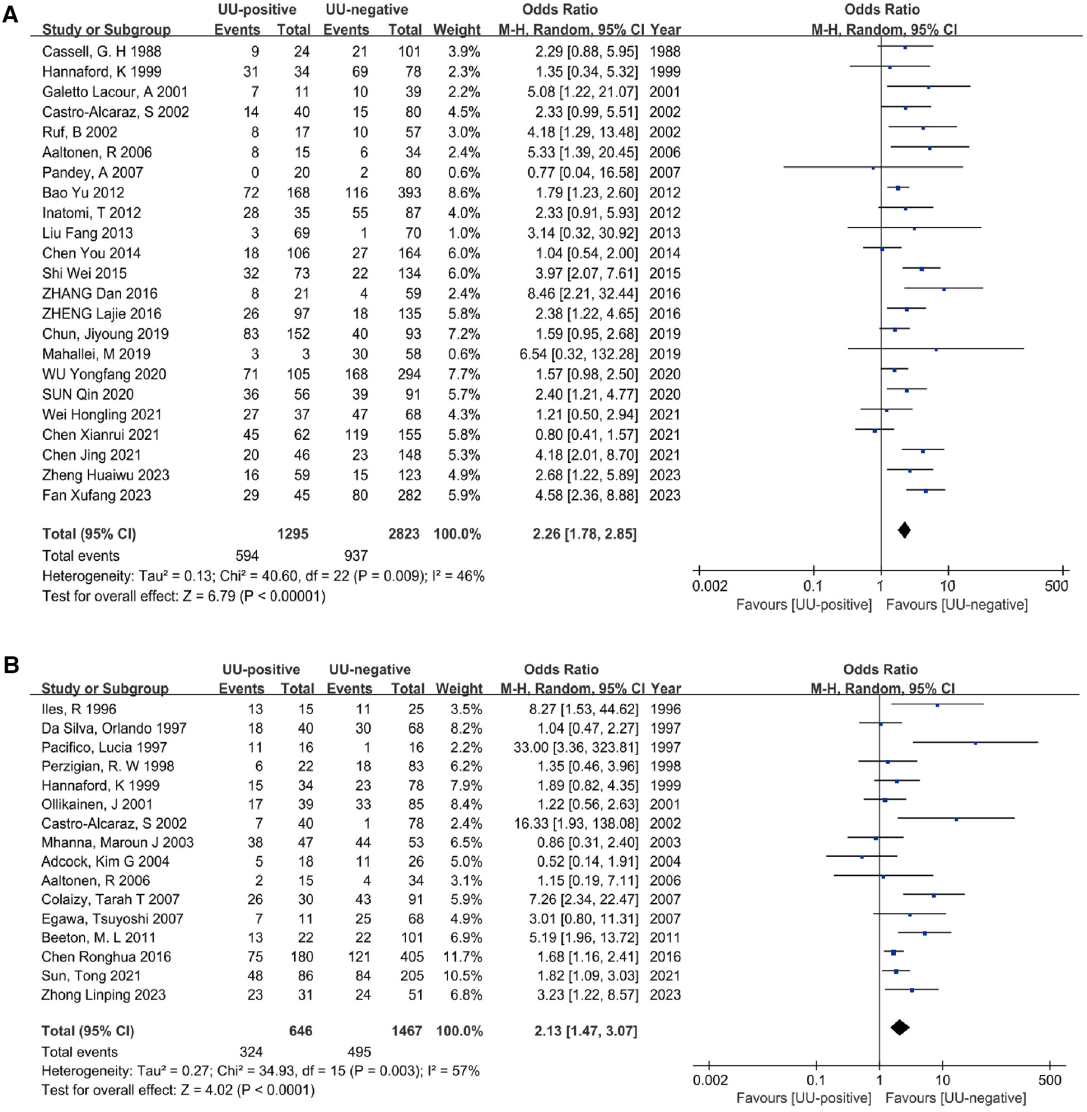
Meta-analyses (**A**). Forest plot of meta-analysis conducted with a diagnosis of BPD at 28 days after birth as an outcome, and the results of the meta-analysis were tested using a random-effects model. (**B**) Forest plot of meta-analysis conducted with a diagnosis of BPD at 36 weeks postmenstrual age as an outcome, and the results of the meta-analysis were tested using a random-effects model.

Twenty-three studies (15, 26–30, 32–37, 39–43, 45, 48, 51, 52, 54, 55) involving a diagnosis of oxygen dependence at 28 days of age included a total of 4,118 preterm infants. The heterogeneity test results indicated no significant heterogeneity among the studies (*I^2^* = 46%, *P *= 0.009). The meta-analysis revealed that UU infection statistically significantly increased the incidence of BPD28 (OR = 2.26, 95% CI: 1.78–2.85). Additionally, the analysis of the outcome was statistically significant (Z = 6.79, *P* < 0.00001) (Figure 3A).

#### 3.4.2 Association between *U. ureaplasma* Colonization and BPD36

The analysis included sixteen studies (25, 31, 38, 44, 46–50, 52, 53, 55–59) (2,113 preterm infants) comparing the association between UU colonization and non-colonization with BPD outcomes at 36 weeks postmenstrual age. There was significant heterogeneity between the two groups (*I^2 ^*= 57%, *P* = 0.003). Using a random effects model, the meta-analysis showed that UU infection statistically significantly increased the incidence of BPD36 (OR = 2.13, 95% CI: 1.47–3.07). Furthermore, the outcome analysis was statistically significant (Z = 4.02, *P* < 0.0001) (Figure 3B).

### 3.5 Subgroup analysis

**Figure 4:**
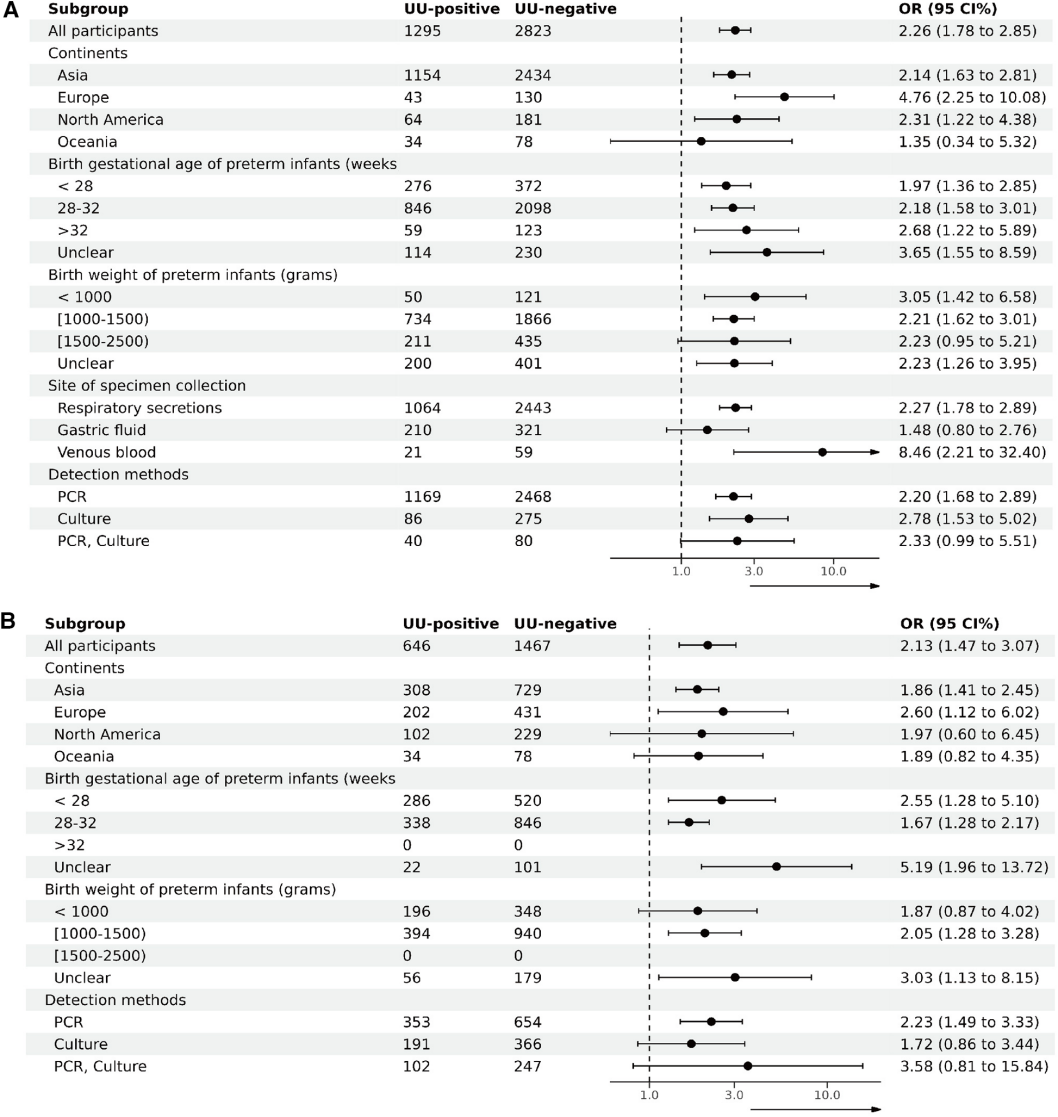
Subgroup analysis (**A**). Subgroup analysis of BPD28 for UU-positive vs. UU-negative. (**B**) Subgroup analysis of BPD36 for UU-positive vs. UU-negative.

To investigate sources of heterogeneity and assess the impact of various factors on outcomes, subgroup analyses of studies targeting BPD28 were conducted based on the continent of the study, the gestational age and birth weight of preterm infants at inclusion, the specimen collection site, and the specimen testing method. Given that all studies involving BPD36 as an outcome used respiratory secretions, subgroup analyses were performed based on the study continent, the gestational age and birth weight of preterm infants at inclusion, and the testing method of the specimens. Subgroup analyses based on continent of inclusion in the study, birth gestational age of preterm infants at the time of inclusion, birth weight at the time of inclusion, site of specimen collection, and method of testing the specimen did not show any evidence of effect modification (Figure 4). Supplementary File S8 details the participants in each subgroup, the results of the heterogeneity test, and the OR and *P* values.

### 3.6 Certainty of evidence

The GRADE methodology was utilized to assess the certainty of the outcomes. In 23 studies diagnosing BPD at 28 days of age, the evidence's certainty was found to be very low. Likewise, in 16 studies diagnosing BPD at 36 weeks postmenstrual age, the evidence's certainty was also deemed very low. The main reasons for the downgrading of the level of evidence were imprecision of the evidence due to the non-randomized controlled cohort design, inconsistency of results across studies, and publication bias. The results and assessments are detailed in Supplementary File S9.

### 3.7 Publication bias and sensitivity analysis

Funnel plots and Egger's test were utilized to evaluate the potential for publication bias. Asymmetry was detected during the visual inspection of the BPD28 and BPD36 funnel plots (Supplementary File S10). However, the Egger test indicated that there was no significant publication bias (BPD28: *P* = 0.1306 > 0.05; BPD36: *P* = 0.0568 > 0.05). Sensitivity analyses were performed for the outcomes (incidence of BPD28 and BPD36) of UU-positive vs. UU-negative. The sensitivity analyses revealed only minor differences between the combined effect values and the total combined effect values, indicating the stability of our results (Supplementary File S11).

## 4 Discussion

UU colonization has been implicated in BPD; however, the conclusions drawn from existing literature lack consistency. This systematic review and meta-analysis synthesizes evidence indicating a significant, positive association between UU colonization and the diagnosis of BPD in preterm infants at both 28 days post-birth and 36 weeks postmenstrual age. Subgroup analyses reveal that the positive correlation between UU colonization and both BPD28 and BPD36 diagnoses persists across various factors, including continent of study, gestational age, birth weight, specimen collection site, and testing method. According to the GRADE assessment, the evidence supporting an association between UU colonization and BPD diagnoses at both 28 days and 36 weeks postmenstrual age is of very low certainty.

Concerning the collection of specimens for UU testing, the majority of current literature identifies tracheal secretions as the optimal specimen type. Among the 36 original studies included in this analysis, 75% (27/36) utilized tracheal secretions as the source of specimens for testing. Lower respiratory secretions exhibit greater diagnostic value for UU infections in preterm infants. However, in clinical settings, the collection of lower respiratory secretions is often constrained in infants without tracheal intubation, potentially resulting in a study cohort characterized by younger gestational ages and lower body weights, factors that heighten disease susceptibility. In this study, specimens were collected within 1 or 3 days of birth in 83.3% (30/36) of the articles, potentially leading to underdiagnosis and associated bias. Mandy et al. (60)collected secretions from four sites - oral, nasal, gastric and tracheal - from preterm infants at 24 to 34 weeks of gestation on days 1–2 and 7–10 postnatal days, respectively. The study concluded that optimal UU detection in preterm infants with a gestational age of 24 to 34 weeks requires collecting cultures from nasal and oral secretions both early (days 1–2) and late (days 7–10) within the first ten days of life. With advancements in neonatal resuscitation techniques, including antenatal glucocorticoids, postnatal lung surfactants, and noninvasive assisted ventilation, exploring alternative sites for UU detection beyond the trachea becomes essential (61, 62). There are studies on the relationship between UU infection and the development of BPD in specimens such as gastric fluid, venous blood, umbilical cord blood, and urine; these methods are much less invasive to the child, and more high-quality clinical studies could be conducted in the future to determine their predictive value (31, 37, 40, 41, 43, 44, 46, 53).

In addition to the test sample and collection time affecting the UU test positivity rate, the test method also significantly impacts the results. The primary techniques for detecting UU infection currently available are culture and PCR. Culture remains the gold standard for UU detection; however, because UU lacks a cell wall and is sensitive to drying and high temperatures, false-negative results are likely if the specimen is collected in insufficient quantities, not sent promptly for testing, or improperly preserved (63, 64). With advancements in detection technology, PCR has become more commonly used in clinical practice, aligning with the data from our included studies (PCR detection alone: 63% (23/36); culture detection alone: 27.8% (10/36); PCR and culture: 8.3% (3/36)). PCR is less time-consuming, more sensitive, and more specific than culture (47, 65). However, the PCR technique may produce false positives; thus, combining it with culture methods or exploring new techniques is necessary to improve test accuracy.

UU is a common conditionally pathogenic microorganism that colonizes the maternal genitourinary tract. UU colonizes 40% to 80% of adult women's vaginas, and the positive test rate in the reproductive tracts of pregnant women can be as high as 82% (66–69). UU colonization is strongly correlated with infertility, spontaneous abortion, chorioamnionitis, premature rupture of membranes, and reproductive tract inflammation (66–69). Perinatal UU is primarily transmitted vertically from mother to child, including through intrauterine and intrapartum infections (70). A study revealed that UU was present in the reproductive tracts of approximately 20% to 25% of pregnant women (71). Mothers can easily transmit pathogens to their newborns if they have UU colonization of the reproductive tract. Kafetzis et al. (72) found that transmission from mothers with colonized vaginal *Ureaplasma* to their newborns was 17% in term infants, 33% in preterm infants, and up to 60% in infants with a birth weight of less than1,000 g. Rittenschober-Boehm et al. (11) found that nearly one-third of pregnant women delivering at ≤32 weeks' gestational age developed ascending Ureaplasma infections after previous vaginal colonization, with a particularly high prevalence among those delivering at <28 weeks' gestational age. Increasing evidence suggests that neonates infected in this way are at risk for developing bronchopulmonary dysplasia (BPD) (66). And maternal *Ureaplasma* colonization was positively associated with BPD (OR 2.4; 95% CI 1.7–3.3) in a recent meta-analysis (73).

The pathogenesis of BPD remains unclear and multifactorial. Potential contributing factors include, but are not limited to, immaturity, volutrauma, oxygen toxicity, mechanical ventilation-induced lung injury, and infection/inflammation (46, 74). During the perinatal period, UU infection can affect normal lung development through several mechanisms. First, UU infection can cause fetal and perinatal activation of the immune system, resulting in the production of cytokines and chemical mediators that interfere with alveolar cell proliferation and differentiation (66, 70). Second, toxins produced by UU may damage lung tissue, leading to impaired cell development (75). Furthermore, persistent chronic infection with UU may result in dysregulation of the host immune response, exacerbating lung inflammation and impeding normal alveolar development (76). Evidence from experimental infection models indicates that pulmonary *Ureaplasma* has proinflammatory and profibrotic effects that contribute to the development of BPD, either independently or in conjunction with other inflammatory stimuli, such as hyperoxia or mechanical ventilation (77). Several clinical studies have also found that preterm infants in the UU-positive group require invasive ventilators for longer periods of time (25, 31, 32).

Our meta-analysis included 5,991 infants, representing the largest sample to date analyzing the association of UU colonization with BPD. This is the first review to include UU data related to BPD that meets the 2001 NICHD and later diagnostic criteria for BPD. The meta-analysis results showed that UU colonization was significantly associated with the development of BPD in preterm infants. Four previous meta-analyses showed evidence of an association between lung colonization with UU and BPD (14, 16–18), Zheng et al.'s meta-analysis included only 11 original studies and was the only one that did not report a significant association (18). The possible reason for not reporting a significant association is that Zheng et al. included only original studies on BPD36 outcomes and included a small sample size (18). Subgroup analysis revealed that differences in gestational age of preterm infants, specimens sent for testing, and testing methods had no effect on the association between intrapulmonary Ureaplasma colonization and BPD, consistent with the results of the meta-analysis by Lowe et al. in 2014 (17). Compared to previous reviews and meta-analyses, this study incorporated additional subgroup analyses, including continent and birth weight of preterm infants. These analyses revealed that differences in these subgroup factors had no effect on the relationship between UU colonization and BPD28 or BPD36, but needs to be repeatedly validated and confirmed in more prospective cohort studies.

This systematic review has some limitations: (1) The studies included in the analysis span 13 countries, encompassing a diverse range of healthcare settings; (2) The publication period of the included studies spans 1988 to 2023, an era marked by significant advances in neonatal medicine, which affect respiratory outcomes in preterm infants; (3) The included studies may have potential publication bias; (4) The majority of studies are not adjusted for confounding variables, potentially resulting in low-quality evidence when combined; (5) False-negative control group data may be present in the included retrospective cohort studies, potentially affecting the accuracy of the results.

Despite these limitations, this study has several strengths: (1) Compared to previous meta-analyses, it provides a clear definition of the diagnosis of BPD; (2) The included studies were all cohort studies, with 52.8% (19/36) being prospective. These studies exhibited a high degree of similarity in terms of the population recruited, the manner of exposure assessment, and the definition of outcomes; (3) 97% of the studies in the meta-analysis were of high quality, with NOS scores of 7–9; (4) The GRADE methodology was used to assess the certainty of evidence.

## 5 Conclusions

This systematic review and meta-analysis suggests that UU colonization is associated with an increased risk of BPD in preterm infants at both 28 days of age and at 36 weeks postmenstrual age (the certainty of the evidence is very low; 36 studies, 5,991 participants). Given the very low level of evidence for the current results, future studies should prioritize well-designed, large-scale, high-quality randomized controlled trials (RCTs) to comprehensively assess the impact of UU colonization on BPD in preterm infants. In the meantime, high-quality randomized controlled studies should be conducted to investigate whether prevention or treatment of UU infections can reduce the risk of BPD.

## Data Availability

The original contributions presented in the study are included in the article/Supplementary Material, further inquiries can be directed to the corresponding authors.
