# Supplementary material for: Association between Ureaplasma urealyticum colonization and bronchopulmonary dysplasia in preterm infants: a systematic review and meta-analysis
Source: Front Pediatr. 2024 Aug 8;12:1436568. doi: 10.3389/fped.2024.1436568 (PMC11338929; doi:10.3389/fped.2024.1436568)
Supplement: Supplementary file 1 [file Datasheet1.pdf]

Supplementary Material

Contents of Supplementary File:

Supplementary File S1. PRISMA Checklist for this systematic review and meta-analysis.
Supplementary File S2. Search strategies.
Supplementary File S3. Eligibility criteria during selection
Supplementary File S4. Criteria for grading methodological quality
Supplementary File S5. Basis for grouping in sub-analysis
Supplementary File S6. List of references with final exclusion reasons
Supplementary File S7. The NOS to rate the risk of bias in cohort and case-control studies
Supplementary File S8. Subgroup analysis of BPD for UU-positive vs. UU-negative
Supplementary File S9. Evidence profiles
Supplementary File S10. Funnel plot
Supplementary File S11. Sensitivity analyses by removing studies one by one for the pooled estimations

Supplementary File S1. PRISMA Checklist for this systematic review and meta-analysis.

Section and Topic	Item #	Checklist item	Location where item is reported
**TITLE**			
Title	1	Identify the report as a systematic review.	1
**ABSTRACT**			
Abstract	2	See the PRISMA 2020 for Abstracts checklist.	1
**INTRODUCTION**			
Rationale	3	Describe the rationale for the review in the context of existing knowledge.	2
Objectives	4	Provide an explicit statement of the objective(s) or question(s) the review addresses.	2
**METHODS**			
Eligibility criteria	5	Specify the inclusion and exclusion criteria for the review and how studies were grouped for the syntheses.	2,3
Information sources	6	Specify all databases, registers, websites, organisations, reference lists and other sources searched or consulted to identify studies. Specify the date when each source was last searched or consulted.	2
Search strategy	7	Present the full search strategies for all databases, registers and websites, including any filters and limits used.	2
Selection process	8	Specify the methods used to decide whether a study met the inclusion criteria of the review, including how many reviewers screened each record and each report retrieved, whether they worked independently, and if applicable, details of automation tools used in the process.	3
Data collection process	9	Specify the methods used to collect data from reports, including how many reviewers collected data from each report, whether they worked independently, any processes for obtaining or confirming data from study investigators, and if applicable, details of automation tools used in the process.	3
Data items	10a	List and define all outcomes for which data were sought. Specify whether all results that were compatible with each outcome domain in each study were sought (e.g. for all measures, time points, analyses), and if not, the methods used to decide which results to collect.	2,3
	10b	List and define all other variables for which data were sought (e.g. participant and intervention characteristics, funding sources). Describe any assumptions made about any missing or unclear information.	2,3
Study risk of bias assessment	11	Specify the methods used to assess risk of bias in the included studies, including details of the tool(s) used, how many reviewers assessed each study and whether they worked independently, and if applicable, details of automation tools used in the process.	3
Effect measures	12	Specify for each outcome the effect measure(s) (e.g. risk ratio, mean difference) used in the synthesis or presentation of results.	3
Synthesis methods	13a	Describe the processes used to decide which studies were eligible for each synthesis (e.g. tabulating the study intervention characteristics and comparing against the planned groups for each synthesis (item #5)).	3

Section and Topic	Item #	Checklist item	Location where item is reported
	13b	Describe any methods required to prepare the data for presentation or synthesis, such as handling of missing summary statistics, or data conversions.	3
	13c	Describe any methods used to tabulate or visually display results of individual studies and syntheses.	3
	13d	Describe any methods used to synthesize results and provide a rationale for the choice(s). If meta-analysis was performed, describe the model(s), method(s) to identify the presence and extent of statistical heterogeneity, and software package(s) used.	3
	13e	Describe any methods used to explore possible causes of heterogeneity among study results (e.g. subgroup analysis, meta-regression).	3
	13f	Describe any sensitivity analyses conducted to assess robustness of the synthesized results.	3
Reporting bias assessment	14	Describe any methods used to assess risk of bias due to missing results in a synthesis (arising from reporting biases).	3
Certainty assessment	15	Describe any methods used to assess certainty (or confidence) in the body of evidence for an outcome.	3
**RESULTS**			
Study selection	16a	Describe the results of the search and selection process, from the number of records identified in the search to the number of studies included in the review, ideally using a flow diagram.	3
	16b	Cite studies that might appear to meet the inclusion criteria, but which were excluded, and explain why they were excluded.	3
Study characteristics	17	Cite each included study and present its characteristics.	3,4
Risk of bias in studies	18	Present assessments of risk of bias for each included study.	8
Results of individual studies	19	For all outcomes, present, for each study: (a) summary statistics for each group (where appropriate) and (b) an effect estimate and its precision (e.g. confidence/credible interval), ideally using structured tables or plots.	4,8
Results of syntheses	20a	For each synthesis, briefly summarise the characteristics and risk of bias among contributing studies.	4,8
	20b	Present results of all statistical syntheses conducted. If meta-analysis was done, present for each the summary estimate and its precision (e.g. confidence/credible interval) and measures of statistical heterogeneity. If comparing groups, describe the direction of the effect.	4,8
	20c	Present results of all investigations of possible causes of heterogeneity among study results.	8
	20d	Present results of all sensitivity analyses conducted to assess the robustness of the synthesized results.	8
Reporting biases	21	Present assessments of risk of bias due to missing results (arising from reporting biases) for each synthesis assessed.	8
Certainty of	22	Present assessments of certainty (or confidence) in the body of evidence for each outcome assessed.	8

Section and Topic	Item #	Checklist item	Location where item is reported
evidence			
**DISCUSSION**			
Discussion	23a	Provide a general interpretation of the results in the context of other evidence.	8
	23b	Discuss any limitations of the evidence included in the review.	11
	23c	Discuss any limitations of the review processes used.	9-11
	23d	Discuss implications of the results for practice, policy, and future research.	9-11
**OTHER INFORMATION**			
Registration and protocol	24a	Provide registration information for the review, including register name and registration number, or state that the review was not registered.	2
	24b	Indicate where the review protocol can be accessed, or state that a protocol was not prepared.	2
	24c	Describe and explain any amendments to information provided at registration or in the protocol.	2
Support	25	Describe sources of financial or non-financial support for the review, and the role of the funders or sponsors in the review.	12
Competing interests	26	Declare any competing interests of review authors.	12
Availability of data, code and other materials	27	Report which of the following are publicly available and where they can be found: template data collection forms; data extracted from included studies; data used for all analyses; analytic code; any other materials used in the review.	12

Supplementary File S2. Search strategies

PubMed

Search Query

#1 (("Bronchopulmonary Dysplasia"[Mesh]) OR ("bronchopulmonary dysplasia*"[Title/Abstract]) OR ("Dysplasia, Bronchopulmonary"[Title/Abstract]) OR ("chronic lung disease"[Title/Abstract]))

#2 (("Ureaplasma urealyticum"[Mesh]) OR ("Ureaplasma urealyticum biovar 2"[Title/Abstract]) OR ("Ureaplasma"[Mesh]) OR (T-Mycoplasma[Title/Abstract]) OR ("Ureaplasma parvum"[Title/Abstract]) OR ("Ureaplasma urealyticum biovar 1"[Title/Abstract]))

#3 #1 AND #2

Embase

No. Query

#1 'lung dysplasia':ti,ab,kw OR 'chronic lung disease of prematurity':ti,ab,kw

#2 'lung dysplasia'/exp OR 'lung dysplasia'

#3 'chronic lung disease of prematurity'/exp OR 'chronic lung disease of prematurity'

#4 'ureaplasma urealyticum':ti,ab,kw OR ureaplasma:ti,ab,kw OR 'ureaplasma urealyticum biovar 2':ti,ab,kw OR 't mycoplasma':ti,ab,kw OR 'ureaplasma parvum':ti,ab,kw OR 'ureaplasma urealyticum biovar 1':ti,ab,kw

#5 'ureaplasma urealyticum'/exp OR 'ureaplasma urealyticum' OR 'ureaplasma'/exp OR ureaplasma

#6 #1 OR #2 OR #3

#7 #4 OR #5

#8 #6 AND #7

Cochrane Library

ID Search

#1 MeSH descriptor: [Bronchopulmonary Dysplasia] explode all trees

#2 ("bronchopulmonary dysplasia")

#3 (chronic lung disease)

#4 MeSH descriptor: [Ureaplasma urealyticum] explode all trees

#5 (Ureaplasma urealyticum)

#6 MeSH descriptor: [Ureaplasma] explode all trees

#7 ("ureaplasma")

#8 #1 OR #2 OR #3

#9 #4 OR #5 OR #6 OR #7

#10 #8 AND #9

Web of Science

ID Search

#1 TS=("Bronchopulmonary Dysplasia" OR "bronchopulmonary dysplasia*" OR "chronic lung disease")

#2 TS=("Ureaplasma urealyticum" OR "Ureaplasma")

#3 #1 AND #2

China National Knowledge Infrastructure (CNKI) - field searching in Chinese

序号 检索表达式

#1 主题：解脲脲原体（精确)

#2 主题：支气管肺发育不良（精确）

#3 **#1 AND #2**

Wanfang Datebase - field searching in Chinese

序号 检索表达式

#1 主题:(解脲脲原体 or 溶脲脲原体 or 解脲支原体) or 全部:(解脲脲原体 or 溶脲脲原体 or 解脲支原体)

#2 主题:(支气管肺发育不良 or 支气管肺发育异常 or 新生儿慢性肺疾病) or 全部:(支气管肺发育不良 or 支气管肺发育异常 or 新生儿慢性肺疾病)

#3 #1 AND #2

China Science and Technology Journal Database (VIP) - field searching in Chinese

序号 检索表达式

#1 题名或关键词：解脲脲原体+溶脲脲原体+解脲支原体

#2 题名或关键词：支气管肺发育不良+支气管肺发育异常+新生儿慢性肺疾病

#3 #1 AND #2

China Biology Medicine disc(CBM) - field searching in Chinese

序号 检索表达式

#1 "解脲脲原体"[加权:扩展] OR "解脲支原体"[全部字段:智能]

#2 "支气管肺发育不良"[加权:扩展] OR "支气管肺发育不良"[全部字段:智能] OR "新生儿慢性肺疾病"[全部字段:智能] OR "支气管肺发育异常"[全部字段:智能]

#3 #1 AND #2

Supplementary File S3. Eligibility criteria during selection

Supplementary File S3 -A Eligibility Criteria

**1.**	**Study population participants**	infants admitted to the NICU with a length of stay sufficient to observe BPD outcomes
**2.**	**Exposure**	Neonates positive for Ureaplasma urealyticum
**3.**	**Comparison**	Neonates negative for Ureaplasma urealyticum.
**4.**	**Outcomes**	Bronchopulmonary dysplasia(BPD)
**5.**	**Study design**	Cohort or Case-control study

Supplementary File S3 -B Definition of outcomes

**Outcomes**	**Definitions**
Bronchopulmonary dysplasia(BPD)	Bronchopulmonary dysplasia diagnosed in newborns still requiring oxygen or respiratory support at 28 days or 36 weeks postmenstrual age

Supplementary File S4. Criteria for grading methodological quality

**Assessment quality of observational studies with the Newcastle-Ottawa Scales (NOS)**	
Low risk of bias	Up to one item was judged inadequate in a study.
Medium risk of bias	Up to three items was judged inadequate in a study.
High risk of bias	More than three items were judged inadequate in a study.
Very high risk of bias	There was no description of methods.

Supplementary File S5. Basis for grouping in sub-analysis

The groups in sub-analysis	Contents in each stratification
**Continents**	
Asia	The countries included in the study belong to the Asian continent
Europe	The countries included in the study belong to the Europe continent
North America	The countries included in the study belong to the North America continent
Oceania	The countries included in the study belong to the Oceania continent
**Birth gestational age of preterm infants (weeks)**	
< 28	The inclusion criteria gestational age < 28 weeks.
28-32	The inclusion criteria gestational age greater than 28 weeks but not more than 32 weeks.
>32	The inclusion criteria gestational age greater than 32 weeks but less than 37 weeks.
Unclear	The inclusion criteria gestational age less than 37 weeks but unclear.
**Birth weight of preterm infants (grams)**	
< 1000	The inclusion criteria birth weight < 1000 g.
[1000-1500)	The inclusion criteria birth weight greater than or equal to 1000g but less than 1500g.
[1500-2500)	The inclusion criteria birth weight greater than or equal to 1500g but less than 2500g.
Unclear	The inclusion criteria birth weight less than or equal to 2500g but the exact range is unclear.
**Site of specimen collection**	
Respiratory secretions	Sample collection specimen sites included nasopharyngeal secretions, pharyngeal swabs, endotracheal aspirates, lung lavage, and other respiratory secretions.
Gastric fluid	The sample collection specimen is gastric fluid.
Venous blood	The sample collection specimen is venous blood.
**Detection methods**	
PCR	The laboratory test method is culture.
Culture	The laboratory test method is polymerase chain reaction.
PCR, Culture	The laboratory test methods are polymerase chain reaction and culture.

Supplementary File S6. List of references with final exclusion reasons

No.	Studies	First author	Reasons of exclusion	Publication year
1.	Association between colonization of the respiratory tract with Ureaplasma species and bronchopulmonary dysplasia in newborns with	Gobec, Katja	Incomplete data	2023

	extremely low gestational age: a retrospective study			
**2.**	Clinical characteristics of preterm and term infants with Ureaplasma in gastric fluid	Abe, Y.	Lack of control group	2023
**3.**	Association of mycoplasma and ureaplasma respiratory colonization and bronchopulmonary dysplasia in extremely preterm infants- a propensity score matched case-control study	Vu, B.	Non-original research	2023
**4.**	Study on the correlation between Ureaplasma urealyticum and bronchopulmonary dysplasia in premature infants	WEN Guo	Diagnostic criteria for BPD did not meet inclusion criteria	2022
**5.**	Clinical study on the relationship between Ureaplasma urealyticum infection and neonatal diseases in 291 preterm infants	Sun, Tong	Overlapping data	2022
**6.**	Impacts of ureaplasma urealyticum infection on respiratory system of pre-mature infants with gestational age less than 32 weeks	Chen, Jing	Incomplete data	2021
**7.**	Bronchopulmonary dysplasia in very preterm infants: Outcome up to preschool age, in a single center of Austria	Reiterer, Friedrich	Imprecise study design	2019
**8.**	Perinatal ureaplasma exposure is associated with increased risk of late onset sepsis and imbalanced inflammation in preterm infants and may add to lung injury	Glaser, K.	The ureaplasma-positive group is positive for Ureaplasma parvum or U. urealyticum	2019
**9.**	Influencing factors of neonatal U. urealyticum respiratory tract colonization and pneumonia	HU Hong-bo	Study population did not meet inclusion criteria	2019
**10.**	The Effect of Macrolide Therapy on Bronchopulmonary Dysplasia in Ureaplasma-Positive Very Low Birth Weight Infants	Kim, Soo Hyun	The ureaplasma-positive group is positive for Ureaplasma parvum or U. urealyticum	2018
**11.**	Clinical analysis of respiratory tract ureaplasma urealyticum infection in preterm infants	Chen, Chao	The definition of BPD is not provided	2017
**12.**	Bronchopulmonary dysplasia in very and extremely low birth weight infants - analysis of selected risk	Kicinski, Przemyslaw	Inability to access full text	2017

	factors			
**13.**	Influence of Ureaplasma urealyticum on the pathogenesis of bronchopulmonary dysplasia in premature infants	Xiong Xiaoyu	Inaccurate study design	2017
**14.**	Analysis of the Relationship between Premature Respiratory Secretions of Ureaplasma Urea Original Body and Neonatal Lung Injury	FAN Li—ying	The definition of BPD is not provided	2016
**15.**	Correlation of Ureaplasma urealyticum Infection with Bronchopulmonary Dysplasia in Very Low Birth Weight Infants with Combined Respiratory Distress Syndrome	Zhao Sai	Inaccurate study design	2015
**16.**	Correlation analysis of preterm infection of ureaplasma urealyticum with bronchopulmonary dysplasia	YANG Yaofeng	Diagnostic criteria for BPD did not meet inclusion criteria	2015
**17.**	Relationship between ureaplasma urealyticum infection and preterm children with bronchopulmonary dysplasia	FANG Xiaoyan	Diagnostic criteria for BPD did not meet inclusion criteria	2014
**18.**	Study on relationship between ureaplasma urealyticum in the respiratory tract of very low birth weight infants and lung injury	CHEN You	Overlapping data	2014
**19.**	Association of Ureaplasma urealyticum infection with bronchopulmonary dysplasia in very low birth weight infants with respiratory distress syndrome	CHEN You	Overlapping data	2014
**20.**	Investigation of respiratory colonization of premature infants with Ureaplasma urealyticum and its association with respiratory disease	Xiao Zhengxiang	Inaccurate study design	2012
**21.**	Association of Positive Ureaplasma in Gastric Fluid with Clinical Features in Preterm Infants	Eugene Jung	Inability to access full text	2011
**22.**	Clarithromycin in preventing bronchopulmonary dysplasia in Ureaplasma urealyticum-positive preterm infants	Ozdemir, R.	Inaccurate study design (placebo-controlled, prospective, randomized study)	2011
**23.**	Association of development of chronic lung disease	Yada, Y.	Inaccurate study design	2010

	of newborns with neonatal colonization of Ureaplasma and cord blood interleukin-8 level			
**24.**	The presence of Ureaplasma urealyticum and Mycoplasma honinis in umbilical cord and the association with the outcome of premature infant	Chang, Shao-hong	Inaccurate study design	2010
**25.**	Association between Ureaplasma urealyticum Colonization and Adverse Outcomes in Premature Infants	Jinsang Yoon	Inability to access full text	2009
**26.**	Association of gastric fluid microbes at birth with severe bronchopulmonary dysplasia	Oue, S.	Comparison between Microbe-negative group and Microbe-positive group	2009
**27.**	The Alabama Preterm Birth Study: Umbilical cord blood Ureaplasma urealyticum and Mycoplasma hominis cultures in very preterm newborn infants	Goldenberg, R. L.	Exposure factors include Ureaplasma urealyticum and Mycoplasma hominis.	2008
**28.**	Maternal genital colonization with Ureaplasma urealyticum promotes preterm delivery: association of the respiratory colonization of premature infants with chronic lung disease and increased mortality	Kafetzis, D. A.	Diagnostic criteria for BPD did not meet inclusion criteria	2004
**29.**	Infection with Ureaplasma urealyticum: Is there a specific clinical and radiological course in the preterm infant?	Theilen, U.	The outcome is not BPD	2004
**30.**	Pulmonary Ureaplasma urealyticum Is Associated with the Development of Acute Lung Inflammation and Chronic Lung Disease in Preterm Infants	Kotecha, S.	Inaccurate study design	2004
**31.**	Identification and quantification of ureaplasmas colonizing the respiratory tract and assessment of their role in the development of chronic lung disease in preterm infants	Heggie, A. D.	Incomplete data	2001
**32..**	Perinatal Ureaplasma urealyticum infection increases the need for hospital treatment during the first year of life in preterm infants	Ollikainen, J.	Diagnostic criteria for BPD did not meet inclusion criteria	2000
**33.**	Ureaplasma urealyticum and its association with chronic lung disease in Asian neonates	Agarwal, P.	Diagnostic criteria for BPD did not meet inclusion criteria	2000

**34.**	Ureaplasma colonisation and chronic lung disease in neonates -: Response	Gilbert, G. L.	Non-original research	2000
**35.**	Impact on the interpretation of confounding variables of the association of Ureaplasma urealyticum (Uu) colonization and the development of chronic lung disease (CLD) among very-low birth-weight (VLBW) infants. comparison of analyses by patterns of colonization vs Uu+/Un- categories	Castro-Alcaraz, S.	Inability to access full text	2000
**36.**	Ureaplasma colonisation and chronic lung disease in neonates	Bhandari, V.	Non-original research	2000
**37.**	Neonatal colonization by Ureaplasma urealyticum and the development of bronchopulmonary dysplasia	Acosta, B.	Inability to access full text	1999
**38.**	Clinical relevance of <i>Ureaplasma urealyticum</i> colonization in preterm infants	Ollikainen, J.	The outcome is not BPD	1998
**39.**	Ureaplasma urealyticum colonization and bronchopulmonary dysplasia: a comparative prospective multicentre study	Abele-Horn, M.	Diagnostic criteria for BPD did not meet inclusion criteria	1998
**40.**	Role of Ureaplasma urealyticum and Chlamydia trachomatis in lung disease in low birth weight infants	Garland, Suzanne M.	Diagnostic criteria for BPD did not meet inclusion criteria	1996
**41.**	Ureaplasma urealyticum tracheal colonization and respiratory disease in newborns	Cordero, L.	Inability to access full text	1996
**42.**	Airway colonization with gram negative bacilli (GNB): Association with severity of bronchopulmonary dysplasia (BPD)	Cordero, Leandro	Inability to access full text	1996
**43.**	Neonatal Ureaplasma urealyticum colonization and chronic lung disease	Jonsson, B.	Diagnostic criteria for BPD did not meet inclusion criteria	1994
**44.**	Chronic lung disease of preterm infants in Finland is not associated with Ureaplasma urealyticum colonization	Saxen, H.	Diagnostic criteria for BPD did not meet inclusion criteria	1993

**45.**	Ureaplasma urealyticum and chronic lung disease	Smyth, A. R.	Incomplete data	1993
**46.**	New prospective studies of the association of Ureaplasma urealyticum colonization and chronic lung disease	Payne, N. R.	Incomplete data	1993
**47.**	Ureaplasma urealyticum infection associated with acute respiratory insufficiency and death in premature infants	Ollikainen, J.	The outcome is not BPD	1993
**48.**	Ureaplasma urealyticum in newborn and premature infants: Association with bronchopulmonary dysplasia	Abele-Horn, M.	Inaccurate study design	1992
**49.**	Respiratory tract colonization with Ureaplasma urealyticum and bronchopulmonary dysplasia in neonates in southern Israel	Horowitz, S.	Diagnostic criteria for BPD did not meet inclusion criteria	1992
**50.**	Role of Ureaplasma urealyticum and other pathogens in the development of chronic lung disease of prematurity	Wang, E. E.	Inability to access full text	1988
**51.**	Ureaplasma urealyticum colonization and chronic lung disease in low birth weight infants	Sanchez, P. J.	Inability to access full text	1988

Supplementary File S7. The NOS to rate the risk of bias in cohort and case-control studies

**References**	**Selection**				**Comparability**	**Outcomes**			**Total (max score: 9)**
	**Representative-ness of the exposed cohort**	**Selection of the non-exposed cohort**	**Ascertainment of exposure to implants**	**Demonstration that outcome of interest was not present at start of study**	**Comparability of cohorts on the basis of the design or analysis**	**Assessment of outcome**	**Was follow up long enough for outcomes to occur**	**Adequacy of follow up of cohorts**	
**Cassell, G. H.1988**	★	★	★	★	NO	★	★	★	7
**Iles, R.1996**	★	★	★	★	★	★	★	★	8
**DaSilva,Orlando**	★	★	★	★	NO	★	★	★	7

**1997**									
**Pacifico, Lucia1997**	★	★	★	★	★	★	★	★	8
**Perzigian,R.W.1998**	★	★	★	★	★	★	★	★	8
**Hannaford, K.1999**	★	★	★	★	★	★	★	NO	7
**Galetto Lacour, A.2001**	★	★	★	★	★	★	★	★	8
**Ollikainen, J.2001**	★	★	★	★	★	★	★	★	8
**Castro-Alcaraz, S.2002**	★	★	★	★	NO	★	★	★	7
**Ruf, B2002.**	★	★	★	★	★	★	★	★	8
**Mhanna, M. J.2003**	★	★	★	★	★★	★	★	★	9
**Adcock, K. G.2004**	★	★	★	★	NO	★	★	★	7
**Aaltonen, R.2006**	★	★	★	★	NO	★	★	★	7
**Colaizy, T. T. 2007**	★	★	★	★	★	★	★	★	8
**Egawa, Tsuyoshi2007**	★	★	★	★	NO	★	★	★	7
**Pandey, A.2007**	★	★	★	★	NO	★	★	★	7
**Beeton, M. L.2011**	★	★	★	★	Not described	★	★	★	7
**Inatomi, T.2012**	★	★	★	★	NO	★	★	★	7
**BAO Yu2012**	★	★	★	★	★	★	★	★	8
**Liu Fang2013**	★	★	★	★	★	★	★	★	8
**Chen You2014**	★	★	★	★	NO	★	★	★	7
**Shi Wei2015**	★	★	★	★	NO	★	★	★	7
**Chen Ronghua2016**	★	★	★	★	★	★	★	★	8
**ZHANG Dan2016**	★	★	★	★	★	★	★	★	8
**ZHENG Lajie2016**	★	★	★	★	NO	★	★	★	7
**Chun, Jiyoung2019**	★	★	★	★	★	★	★	★	8
**Mahallei, M.2019**	★	★	★	★	NO	★	★	NO	6
**WU Yongfang2020**	★	★	★	★	NO	★	★	★	7
**Sun, Qin2020**	★	★	★	★	★	★	★	★	8
**Chen Jing2021**	★	★	★	★	★	★	★	NO	7
**Chen Xianru2021**	★	★	★	★	NO	★	★	★	7
**Wei Hongling2021**	★	★	★	★	★	★	★	★	8
**Sun, Tong2021**	★	★	★	★	NO	★	★	★	7
**Fan Xufang2023**	★	★	★	★	★	★	★	★	8
**Zheng Huaiwu2023**	★	★	★	★	NO	★	★	★	7
**Zhong Linping2023**	★	★	★	★	★★	★	★	★	9

★ Meet the scoring conditions

Supplementary File S8. Subgroup analysis of BPD for UU-positive vs. UU-negative

A. Subgroup analysis of BPD28 for UU-positive vs. UU-negative

		No. of Participants		Heterogeneity			
	No. of Studies	UU-positive	UU-negative	I^2 (%)	P	OR (95% CI)	P Value
**BPD28**	23	594/1295	937/2823	46	0.009	2.26 (1.78, 2.85)	< 0.00001
**Continents**							
Asia	17	517/1154	806/2434	55	0.004	2.14 (1.63, 2.81)	< 0.00001
Europe	3	23/43	26/130	0	0.96	4.76 (2.25, 10.08)	< 0.00001
North America	2	23/64	36/181	0	0.97	2.31 (1.22, 4.38)	0.01
Oceania	1	31/34	69/78	Not applicable	Not applicable	1.35 (0.34, 5.32)	0.67
**Birth gestational age of preterm infants (weeks)**							
< 28	5	164/276	185/372	0	0.51	1.97 (1.36, 2.85)	0.0003
28-32	14	394/846	711/2098	60	0.002	2.18 (1.58, 3.01)	< 0.00001
>32	1	16/59	15/123	Not applicable	Not applicable	2.68 (1.22, 5.89)	0.01
Unclear	3	20/114	26/230	18	0.30	3.65 (1.55, 8.59)	0.003
**Birth weight of preterm infants (grams)**							
< 1000	2	36/50	61/121	0	0.32	3.05 (1.42, 6.58)	0.004
[1000-1500)	13	370/734	676/1866	51	0.02	2.21 (1.62, 3.01)	< 0.00001
[1500-2500)	3	54/211	65/435	76	0.02	2.23 (0.95, 5.21)	0.06
Unclear	5	134/300	135/401	30	0.22	2.23 (1.26, 3.95)	0.006
**Site of specimen collection**							
Respiratory secretions	19	537/1064	850/2443	43	0.02	2.27 (1.78, 2.89)	< 0.00001
Gastric fluid	3	49/210	83/321	17	0.30	1.48 (0.80, 2.76)	0.21
Venous blood	1	8/21	4/59	Not applicable	Not applicable	8.46 (2.21, 32.4)	0.002
**Detection methods**							
PCR	18	525/1169	812/2468	54	0.003	2.20 (1.68, 2.89)	< 0.00001
Culture	4	55/86	110/275	0	0.50	2.78 (1.53, 5.02)	0.0007
PCR, Culture	1	14/40	15/80	Not applicable	Not applicable	2.33 (0.99, 5.51)	0.05

B. Subgroup analysis of BPD36 for UU-positive vs. UU-negative

		No. of Participants		Heterogeneity			
	No. of Studies	UU-positive	UU-negative	I^2 (%)	P	OR (95% CI)	P Value
**BPD36**	16	324/646	495/1467	57	0.003	2.13 (1.47, 3.07)	< 0.0001
**Continents**							
Asia	4	153/308	254/729	0	0.56	1.86 (1.41, 2.45)	< 0.0001
Europe	8	125/202	169/431	73	0.0005	2.60 (1.12, 6.02)	0.03

North America	3	31/102	49/229	66	0.05	1.97 (0.60, 6.45)	0.27
Oceania	1	15/34	23/78	Not applicable	Not applicable	1.89 (0.82, 4.35)	0.14
**Birth gestational age of preterm infants (weeks)**							
< 28	10	158/286	192/520	68	0.0008	2.55 (1.28, 5.10)	0.008
28-32	5	153/338	281/846	0	0.80	1.67 (1.28, 2.17)	0.001
>32	0	0	0	Not applicable	Not applicable	Not estimable	Not applicable
Unclear	1	13/22	22/101	Not applicable	Not applicable	5.19 (1.96, 13.72)	0.0009
**Birth weight of preterm infants (grams)**							
< 1000	7	125/196	167/348	67	0.006	1.87 (0.87, 4.02)	0.11
[1000-1500)	7	171/394	283/940	52	0.05	2.05 (1.28, 3.28)	0.003
[1500-2500)	0	0	0	Not applicable	Not applicable	Not estimable	Not applicable
Unclear	2	28/56	45/179	58	0.12	3.03 (1.13, 8.15)	0.03
**Detection methods**							
PCR	6	181/353	301/854	35	0.17	2.23 (1.49, 3.33)	< 0.0001
Culture	7	105/191	141/366	61	0.02	1.72 (0.86, 3.44)	0.13
PCR, Culture	3	38/102	53/247	80	0.007	3.58 (0.81, 15.84)	0.09

Supplementary File S9. Evidence profiles

Certainty assessment							№ of patients		Effect		Certainty	Importance
№ of studies	Study design	Risk of bias	Inconsistency	Indirectness	Imprecision	Other considerations	UU (+)	UU (-)	Relative (95% CI)	Absolute (95% CI)		
**BPD28**												
23	non-randomised studies	not serious	very serious	not serious	not serious	publication bias strongly suspected all plausible residual confounding would reduce the demonstrated effect	594/1295 (45.9%)	937/2823 (33.2%)	**OR 2.26** (1.78 to 2.85)	**197 more per 1,000** (from 137 more to 254 more)	⊕◯◯◯ Very low	critical
**BPD36**												
16	non-randomised studies	not serious	serious	not serious	not serious	publication bias strongly suspected all plausible residual confounding would reduce the demonstrated effect	324/646 (50.2%)	495/1467 (33.7%)	**OR 2.13** (1.47 to 3.07)	**183 more per 1,000** (from 91 more to 272 more)	⊕◯◯◯ Very low	critical

CI: confidence interval; OR: odds ratio

Supplementary File S10. Funnel plot

Supplementary File S10-A Funnel plot of BPD28 for UU-positive VS UU-negative

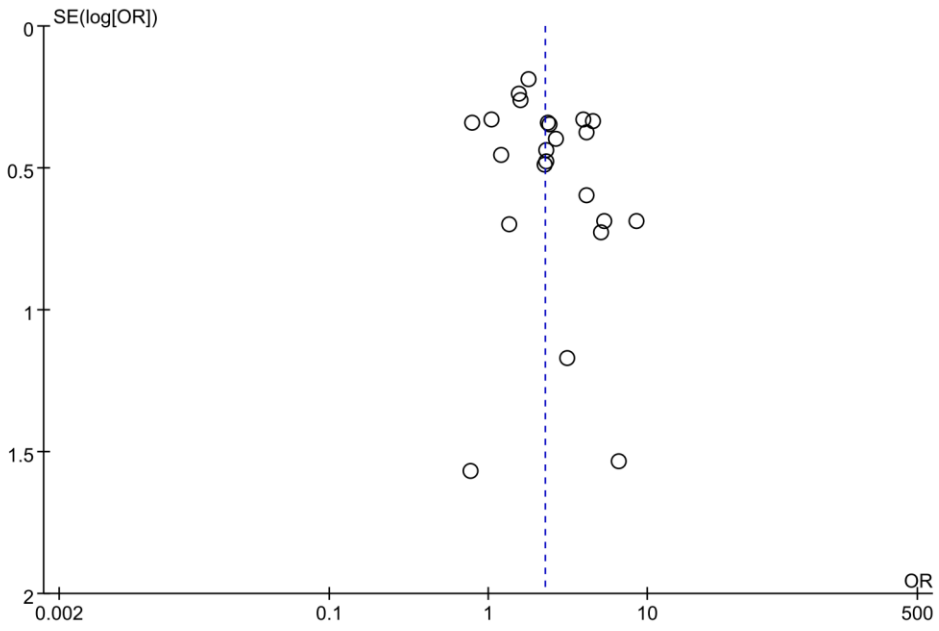

Supplementary File S10-B Funnel plot of BPD36 for UU-positive VS UU-negative

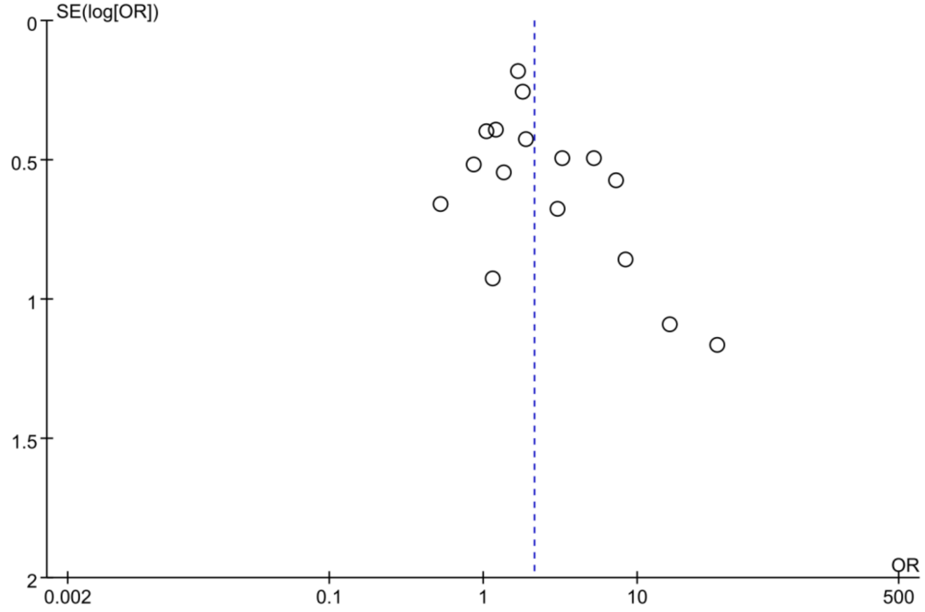

Supplementary File S11. Sensitivity analyses by removing studies one by one for the pooled estimations

Supplementary File S11-A BPD28

**Removing Study ID**	**Summary OR [95% CI]**	**p-value**
Cassell, G. H 1988	2.26 [1.77, 2.89]	**< 0.00001**
Hannaford, K 1999	2.29 [1.80, 2.91]	**< 0.00001**
Galetto Lacour, A 2001	2.21 [1.75, 2.81]	**< 0.00001**
Castro-Alcaraz, S 2002	2.26 [1.77, 2.89]	**< 0.00001**
Ruf, B 2002	2.21 [1.74, 2.81]	**< 0.00001**
Aaltonen, R 2006	2.21 [1.74, 2.79]	**< 0.00001**
Pandey, A 2007	2.27 [1.79, 2.88]	**< 0.00001**
Bao Yu 2012	2.32 [1.79, 3.00]	**< 0.00001**

Inatomi, T 2012	2.26 [1.77, 2.89]	**< 0.00001**
Liu Fang 2013	2.25 [1.77, 2.86]	**< 0.00001**
Chen You 2014	2.36 [1.87, 2.98]	**< 0.00001**
Shi Wei 2015	2.17 [1.71, 2.75]	**< 0.00001**
ZHANG Dan 2016	2.17 [1.73, 2.73]	**< 0.00001**
ZHENG Lajie 2016	2.26 [1.76, 2.90]	**< 0.00001**
Chun, Jiyoung 2019	2.33 [1.81, 2.98]	**< 0.00001**
Mahallei, M 2019	2.24 [1.77, 2.85]	**< 0.00001**
WU Yongfang 2020	2.33 [1.82, 2.99]	**< 0.00001**
SUN Qin 2020	2.26 [1.76, 2.89]	**< 0.00001**
Wei Hongling 2021	2.32 [1.82, 2.95]	**< 0.00001**
Chen Xianrui 2021	2.37 [1.90, 2.95]	**< 0.00001**
Chen Jing 2021	2.17 [1.72, 2.75]	**< 0.00001**
Zheng Huaiwu 2023	2.24 [1.75, 2.87]	**< 0.00001**
Fan Xufang 2023	2.14 [1.70, 2.69]	**< 0.00001**

Supplementary File S11-B BPD36

**Removing Study ID**	**Summary OR [95% CI]**	**p-value**
Iles, R 1996	2.01 [1.40, 2.90]	**0.0002**
Pacifico, Lucia 1997	1.98 [1.40, 2.79]	**0.0001**
Da Silva, Orlando 1997	2.27 [1.54, 3.34]	**< 0.0001**
Perzigian, R. W 1998	2.20 [1.49, 3.25]	**< 0.0001**
Hannaford, K 1999	2.17 [1.46, 3.23]	**0.0001**
Ollikainen, J 2001	2.25 [1.52, 3.33]	**< 0.0001**
Castro-Alcaraz, S 2002	2.01 [1.40, 2.87]	**0.0001**
Mhanna, Maroun J 2003	2.26 [1.55, 3.30]	**< 0.0001**
Adcock, Kim G 2004	2.27 [1.57, 3.27]	**< 0.0001**
Aaltonen, R 2006	2.18 [1.49, 3.18]	**< 0.0001**
Colaizy, Tarah T 2007	1.94 [1.36, 2.77]	**0.0003**
Egawa, Tsuyoshi 2007	2.10 [1.43, 3.08]	**0.0002**
Beeton, M. L 2011	1.97 [1.37, 2.85]	**0.0003**
Chen Ronghua 2016	2.25 [1.46, 3.47]	**0.0002**

Sun, Tong 2021	2.21 [1.45, 3.35]	**0.0002**
Zhong Linping 2023	2.07 [1.41, 3.04]	**0.0002**
